# Experimental and Numerical Analysis of Fracture Mechanics Behavior of Heterogeneous Zones in S690QL1 Grade High Strength Steel (HSS) Welded Joint

**DOI:** 10.3390/ma16216929

**Published:** 2023-10-28

**Authors:** Damir Tomerlin, Dražan Kozak, Luka Ferlič, Nenad Gubeljak

**Affiliations:** 1 Mechanical Engineering Faculty in Slavonski Brod, University of Slavonski Brod, Trg I. B. Mažuranić 2, 35000 Slavonski Brod, Croatia; dkozak@unisb.hr; 2 Faculty of Mechanical Engineering, University of Maribor, Smetanova 17, 2000 Maribor, Slovenia; luka.ferlic@um.si (L.F.); nenad.gubeljak@um.si (N.G.)

**Keywords:** heterogeneous welded joint, high strength steel, mechanical testing, damage, fracture, mechanical properties, finite element analysis

## Abstract

The heterogeneity of welded joints’ microstructure affects their mechanical properties, which can vary significantly in relation to specific weld zones. Given the dimensional limitations of the available test volumes of such material zones, the determination of mechanical properties presents a certain challenge. The paper investigates X welded joint of S690QL1 grade high strength steel (HSS), welded with slightly overmatching filler metal. The experimental work is focused on tensile testing to obtain stress-strain properties, as well as fracture mechanics testing. Considering the aforementioned limitations of the material test volume, tensile testing is carried out with mini tensile specimens (MTS), determining stress-strain curves for each characteristic weld zone. Fracture mechanical testing is carried out to determine the fracture toughness using the characteristic parameters. The experimental investigation is carried out using the single edge notch bend (SENB) specimens located in several characteristic welded joint zones: base metal (BM), heat affected zone (HAZ), and weld metal (WM). Fractographic analysis provides deeper insight into crack behavior in relation to specific weld zones. The numerical simulations are carried out in order to describe the fracture behavior of SENB specimens. Damage initiation and evolution is simulated using the ductile damage material behavior. This paper demonstrates the possibility of experimental and numerical determination of fracture mechanics behavior of characteristic heterogeneous welded joint zones and their influence on crack path growth.

## 1. Introduction

**Figure 1:**
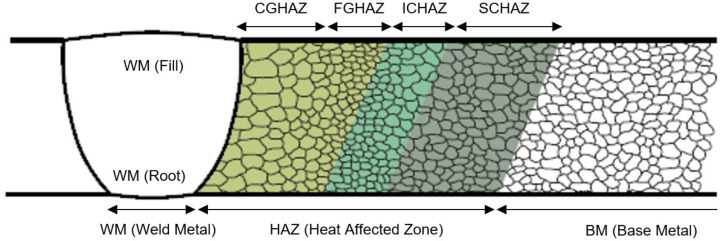
Welded joint—characteristic zones [1].

During the fusion welding process, several characteristic zones are being formed inside the welded joint, namely the base metal (BM), weld metal (WM), and heat affected zone (HAZ). HAZ is considered the part of the base metal that was not being melted during the fusion welding but was exposed to temperature below the melting point (and subsequently cooled). Width of HAZ is generally dependent on the type of welding process and the process parameters. Boundary between WM and HAZ is the fusion line (FL). The HAZ zone further consists of several characteristic segments with different microstructures and mechanical properties. These segments, arranged in the direction from weld metal to base metal, are as follows: the coarse-grained zone (CGHAZ), fine-grained zone (FGHAZ), inter-critical zone (ICHAZ), and sub-critical zone (SCHAZ), shown in Figure 1. Due to such segmentation, welded joints show significant heterogeneity of their microstructures, which also affect their mechanical properties in relation to specific weld zones. Research of fusion weld characteristic zones is already carried out in past studies [1,2,3].

Investigation of S690QL1 steel welded joints exposed to post-weld heat treatment (PWHT) and the resulting influence on specific zones microstructure, mechanical properties, and residual stress has already been carried out by authors Tomerlin et al. [4]. General investigations of local microstructure and mechanical properties of welded joint metal is described by Karkhin [5]. Evaluation of the factors influencing the strength of HSLA steel weld joint with softened HAZ, by means of physical HAZ simulation is carried out by Maurer et al. [6]. Expected mechanical properties of steel weld HAZ zones are given in Table 1. During welding, CGHAZ, near FL, is also heated in high austenite zone, and FGHAZ in lower austenite zone above A_c3_ line. Coarse grains in HAZ grow with increasing temperature and times.

The weld segmentation entails dimensional limitations of the available test volumes of such welded joint material zones, consequently making the determination of material mechanical properties very challenging. This is especially noticeable in HAZ segments. Tensile testing of relatively small material volumes, to obtain stress-strain curves, is already demonstrated using ASTM E8 round tension test small-size proportional specimens [7]. In scope of welded joint testing, such round specimens can capture the BM, WM and only general HAZ properties. The possibility of testing tensile properties along the linear direction (e.g., transverse welded joint direction) is successfully demonstrated by implementation of mini tensile specimens (MTS) [8,9,10,11]. This testing approach enables capturing the HAZ specific segments properties.

According to metallurgical manufacturing processes, there are several categories of HSS steels available, such as quenched and tempered (QT) steel S690QL1 being relevant in this paper. HSS steel grades are intended for structural applications so it is mandatory for them to have good weldability, using common fusion welding processes [12]. It is important to establish welding parameters that lead to least degradation of mechanical properties in heat affected zone (HAZ), which is the most sensitive zone in welded joint. With the steel material strength increase, the permissible limits of welding heat input are drastically reduced and the welding process must be within tolerable limits [12,13,14].

Investigations of crack behavior of welded joint structures in general is a relevant and thus thoroughly researched topic. Fracture mechanics approach, according to ASTM E1820, to obtain the fracture toughness using the characteristic parameters, has found wide application in experimental testing of welded joints. *J*-integral calculation procedure, using the SENB three-point bending specimens, for strength mismatched welds in S690QL high strength low alloyed steel is carried out by Štefane et al. [15]. The relationship between microstructure and cleavage fracture in the most brittle areas of welded S690 high strength steel structures, the coarse-grained and intercritically reheated coarse-grained HAZ (CGHAZ and ICCGHAZ), is investigated by Bertolo et al. [16]. Characteristic HAZ microstructures are generated using thermo-mechanical simulator. Microstructures are than characterised, and the tensile and fracture properties investigated [16]. Investigation of the effect of blunt defects on the structural integrity assessment of S690 steel, using notched tensile and SENB specimens, with various notch acuities are performed by Kim et al. and show an increase in effective ductile fracture toughness with increasing notch radius. [17]. Fracture toughness using a Pipe Ring Notched Bend (PRNB) specimen and Single Edge Notch Bend (SENB) specimen was investigated by Damjanović et al. [18]. Some of the authors investigating the fracture toughness of various high strength steel grades, using the *J*-R curve and the stretch zone methods, are Yu et al. [19], Chai et al. [20], Frómeta et al. [21], and Gao et al. [22].

This paper and its related investigation aim to determine the mechanical properties in scope of heterogeneous zones of welded joint. The X welded joint of S690QL1 grade High Strength Steel (HSS), welded with slightly overmatching filler metal, is investigated. Experimentally determined stress–strain properties, using MTS tensile testing specimens and fracture toughness parameters, using three-point bending SENB specimens, gives the detailed insight into behavior of specific welded joint zones. Numerical simulations of crack behavior are carried out for SENB specimens, using the ductile damage material models. Simulations show good agreement with DIC imagery, giving the confirmation of specific methodology general applicability to welded joints or heterogeneous structures.

## 2. Materials and Experimental Methods

### 2.1. Materials

The base metal (BM) material investigated in this study is High Strength Steel (HSS) S690QL1 grade. The steel is manufactured with Quenching and Tempering (QT) process and has fine-grained microstructure. The 40 mm thick plates are supplied in hot rolled condition, according to EN 10025-6 standard [23]. The same standard declares the yield strength *R*_p0.2_ ≥ 690 MPa, while tensile strength is in the range *R*_m_ = 770–940 MPa for this steel grade, valid for plate thickness up to 50 mm. QL1 corresponds to extra-tough grade with minimum notch toughness values determined at −60 °C [24,25].

In order to produce a welded joint with high mechanical properties and having the ability to withstand high loading during the service life, the corresponding grade of high strength Mn3Ni1CrMo (ER110S-G) filler metal is used [26]. The filler high strength micro- alloyed solid wire is according to EN ISO 16834-A/AWS A5.28 standards, with Weld Metal (WM) declared yield strength *R*_p0.2_ ≈ 800 MPa, also featuring excellent ductility and crack resistance [27,28]. For both these materials, the mechanical properties and chemical composition, as declared in manufacturer certificates, are listed in Table 2 and Table 3.

### 2.2. Welded Joint and Process

**Figure 2:**
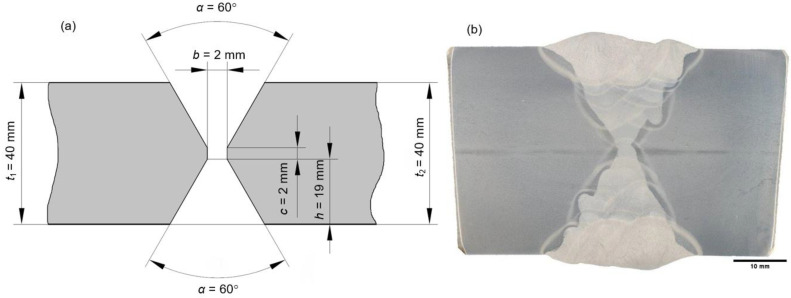
X-welded joint: (**a**) groove geometry and preparation; (**b**) welding passes macrograph.

The test coupon is made from two 40 mm thick steel plates, welded in double V-butt weld (X-welded joint) configuration. Authors used this test coupon for series of different welded joint material testing, some of them published in scope of related investigations and research, therefore the overall welded test coupon length of 1000 mm is needed to accommodate all of the test specimens. Groove preparation is previously performed according to recommendations given in EN ISO 9692-1, for joint Ref. No. 2.5.1 (Figure 2a), machining the whole 1000 mm edges length of 40 mm plates [30]. Welding is carried out using the Gas Metal Arc Welding (GMAW) process with 1.2 mm diameter filler wire, using shielding gas 82%Ar + 18%CO_2_ with 12–16 L/min flow, in multi-pass welding (22 welding passes total) configuration, as shown in macrograph (Figure 2b).

The certain difference in strength material properties between BM and WM is described by the mismatch factor using the equation:

(1) $$M=\frac{\sigma_{YW}}{\sigma_{YB}}$$

where *σ*_YW_ and *σ*_YB_ represent the yield strength of the weld metal (WM) and the yield strength of the base metal (BM), respectively. In this welded joint, mismatch factor *M* = 1.16 (Table 2) which indicates the slightly over-matching (OM) weld metal, with *M* > 1 [8,31]. In general, HSS steel grades can be welded using various matching filler metals. For HSS of very high strength values (*R*_p0.2_ ≥ 1000 MPa), since no strength-matching filler metals are available; general practice is to use filler wires of lower strength, and produce welded joints of under-matching (UM) quality, with *M* < 1.

The welding process is performed according to general HSS welding requirements. Pre-heating temperature is *T*_p_ ≤ 100 °C, while the interpass temperature was limited to *T*_i_ ≤ 180 °C. Welding process is carried out with overall low heat input, averaging *Q* = 0.85 kJ/mm for root passes and *Q* = 0.87 kJ/mm for fill passes. This consequently leads to short Δ*t*_8/5_ HAZ cooling times interval between 800 °C and 500 °C, related to the most significant structural changes inside the steel material. Short cooling times lead to formation of martensitic metallurgical structure and increased hardness along with the risk of brittle fractures [32,33]. The obtained average Δ*t*_8/5_ = 4.4 s for root passes and Δ*t*_8/5_ = 4.5 s for fill passes. The welding process parameters are listed in Table 4.

### 2.3. Test Plan

**Figure 3:**
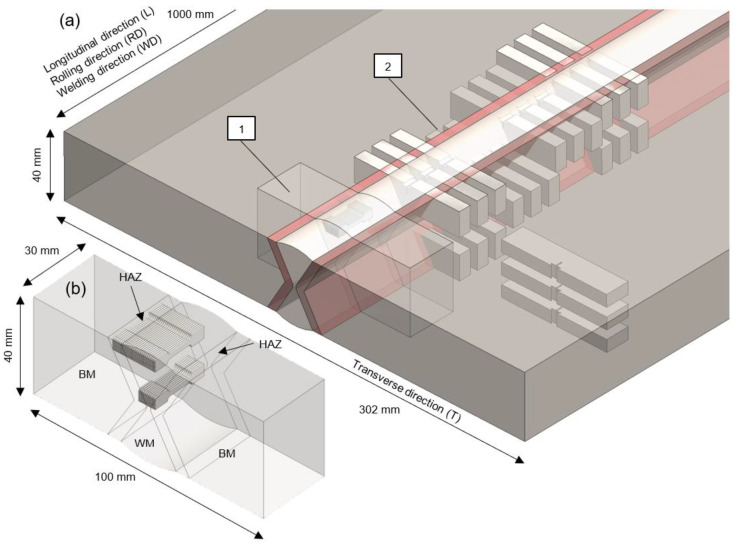
X joint welded test coupon: (**a**) disposition of test specimens; (**b**) MTS subsection.

The welded test coupon is prepared according to the test plan to accommodate all of the specimens required for specific test procedures (Figure 3). Subsections of cuboid shape are prepared for specific groups of testing procedures, enumerated, and finally separated mechanically from the test coupon using a band saw.

The specific testing procedures according to specimen groups (Figure 3):

- 1—Mini tensile specimens (MTS) testing
- 2—Fracture mechanics testing, fractographic analysis

Two main groups of mechanical testing procedures relate to MTS testing to determine tensile and stress-strain properties, and SENB fracture mechanics testing followed by microstructural and fractographic analysis.

### 2.4. Mini Tensile Specimens (MTS) Testing

**Figure 4:**
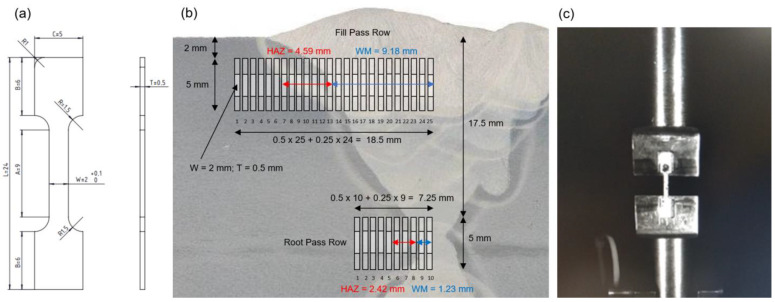
MTS testing: (**a**) specimen geometry; (**b**) specimens positioning along the welded joint transverse direction; (**c**) test setup.

The tensile properties along the transverse welded joint line are determined using two sets of mini tensile specimens (MTS) [8,9,10]. Using this substandard sized tensile specimens is required due to limitations of the material test volume inside the welded joint in order to determine the stress-strain curves for each characteristic weld zone. The first set of MTS specimens is positioned in fill pass material volume (MTS R1–R24 and R25 at centerline) of X welded joint, vertically distanced 2 mm from the top surface of 40 mm steel plate. Second set of MTS specimens is positioned in root pass material volume (MTS C1-9 and C10 at centerline), distanced 17.5 mm from the top surface. Both sets are oriented in the transverse direction including BM, HAZ, and WM up to the joint centerline, as shown in Figure 4b.

The MTS specimens are flat sheet tensile specimens, with specific dimensions *L* = 24 mm, *A* = 9 mm, *B* = 6 mm, *C* = 5 mm, and thickness *T* = 0.5 mm, with geometry shown in Figure 4a. They are manufactured using the electrical discharge wire cutting (EDWC) technology, with the wire diameter 0.25 mm, which also dictates the lateral distance between the test specimens. Uniaxial tensile testing of MTS specimens is performed on Instron 1255 testing machine (Instron, Norwood, MA, USA), at ambient temperature, under a constant stroke velocity of 0.1 mm/min, using stereo-optical system ARAMIS (Carl Zeiss GOM Metrology GmbH, Braunschweig, Germany) with a digital image correlation (DIC) technique, as shown in Figure 4c.

The experimental testing method with MTS specimens enables detailed investigation of tensile properties for main welded joint characteristic zones, as well as specific HAZ segments. The specific locations inside the welded joint, from which the individual MTS specimens are extracted, are listed in Table 5.

### 2.5. Fracture Mechanics Testing

**Figure 5:**
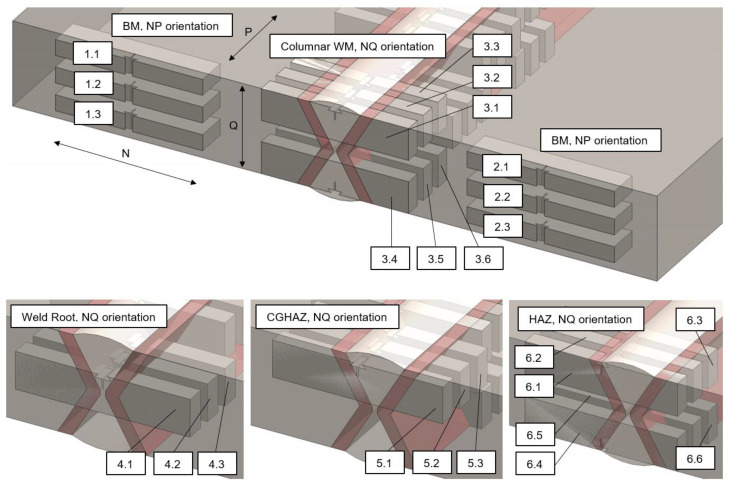
Fracture mechanics testing disposition of SENB specimens inside the welded joint.

Fracture mechanics testing is carried out to investigate all of the characteristic X welded joint zones: base metal (BM), heat affected zone (HAZ), and weld metal (WM). To examine all of the zones of interest, the single edge notch bend (SENB) test specimens are placed inside the welded joint volume. When choosing the specimens positions and notch locations, the authors used the general recommendations given in BS 7448: Part 2 [34]. Such disposition of SENB test specimens inside the X welded joint is shown in Figure 5.

The investigated welded joint zones are related to: BM (specimen groups 1 and 2), columnar WM on weld centre line, upper and lower side of X joint (specimen group 3), WM root of lower side of X joint (specimen group 4), CGHAZ adjacent to columnar weld metal (specimen group 5) and through HAZ volume, upper and lower side of the X joint (specimen group 6). According to BS 7448: Part 2, crack plane orientation code for welded fracture toughness specimens, the codes used on specimens are: NP (crack opening in N direction and crack propagation in P direction) and NQ (crack opening in N direction and crack propagation in Q direction). All of the specimen combinations are listed in specimen disposition matrix, in Table 6.

**Figure 6:**
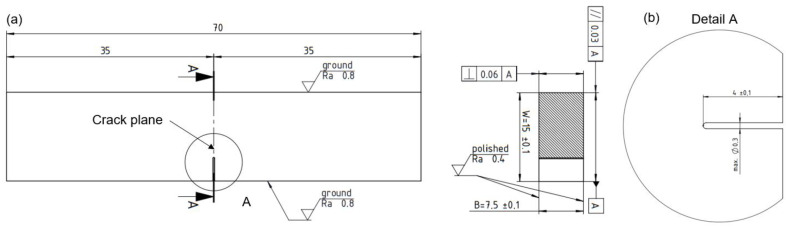
SENB fracture mechanics testing specimen: (**a**) specimen geometry and dimensions; (**b**) fatigue crack starter notch.

The SENB fracture mechanics testing specimens have geometry according to recommendations given in ASTM E1820 [35,36], Single Edge Notch Bend (SENB) Specimen, with specific dimensions *W* = 15 mm, *B* = 7.5 mm and overall specimen length of 70 mm, shown in Figure 6a. The specimens are manufactured by machining, with side surfaces polished and etched using the 3% HNO_3_ nitric acid in ethyl alcohol (Nital) to reveal welded joint characteristic zones and features in order to precisely determine the position of fatigue crack starter notches. The notch geometry according to ASTM E1820, Figure 6 (i.e., ‘Fatigue Crack Starter Notch Configurations’) is chosen to be the narrow notch type. Notches are manufactured using the electrical discharge wire cutting (EDWC) technology, with a wire diameter of 0.3 mm, as shown in Figure 6b.

**Figure 7:**
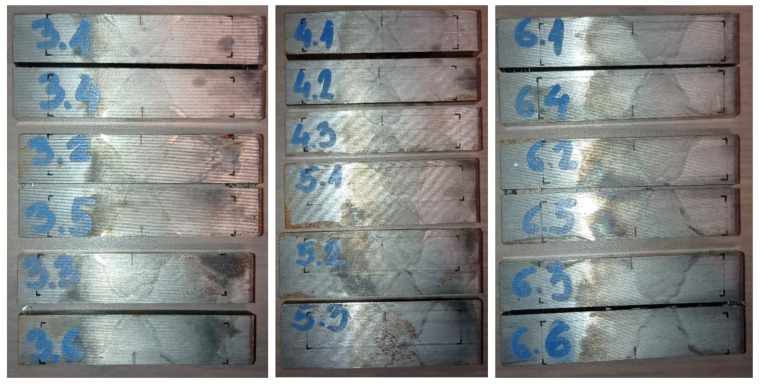
Extracted material volumes with marked SENB specimen placement sketches and fatigue crack starter notch lines.

Prior to final machining, as shown in Figure 7, the sufficient material volume for each SENB specimen is first extracted from the welded test coupon, using the band saw cutting process with low generated heat input. Low heat input is required to avoid thermal degradation of material to be tested. The etched faces reveal the characteristic welded joint features and fusion lines. The sketches of corresponding SENB samples geometry, required for samples manufacturing by machining, are marked with black lines, while notches are marked with red lines to be manufactured by EDWC, shown in Figure 7.

**Figure 8:**
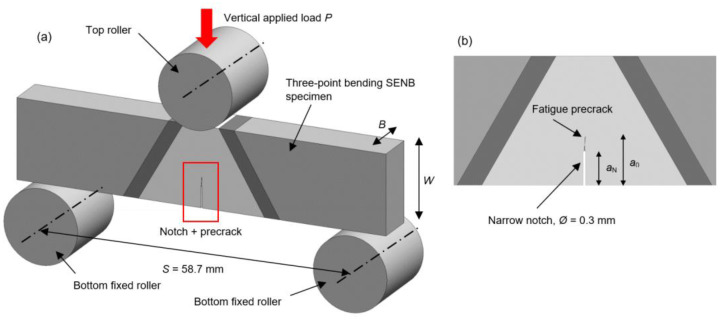
Testing of SENB specimen: (**a**) test setup; (**b**) notch and fatigue precrack.

Since artificially manufactured notches are not able to simulate natural crack well enough, and finally provide the satisfactory fracture toughness test result, the fatigue precracking procedure, according to ASTM E1820 standard, is performed as continuation of EDWC manufactured fatigue crack starter notches [15]. The fatigue precrack is produced by cyclically loading the three-point bending SENB specimens, using servo-hydraulic testing machine INSTRON 1255 (Instron, Norwood, MA, USA), shown in Figure 8. Crack growth is monitored with Keyence VHX digital microscope (Keyence Corp., Itasca, IL, USA). Precracking procedure of SENB specimens is carried out with a load ratio *R* = 0.1 and applied maximum SIF to elastic modulus ratio *K*_max_/*E* ≤ 1.1 × 10^−4^ m^1/2^.

Fatigue precracking specifications with crack dimensions are given in Table 7.

Where *a*_N_ is notch length, *a*_0_ is initial measured fatigue crack (FC) size, *a*_0_/W is fatigue crack size ratio, *a*_p_ is physical fracture crack (PFC) size, *a*_p_/W is physical fracture crack size ratio, and Δ*a* is crack extension. All crack lengths in scope of this research are in range 0.25 ≤ a/W ≤ 0.52. For all samples, the nominal *a*_N_ = 4 mm, while *a*_0_ fatigue crack length is determined in a way that the crack tip enters the specific weld zone material.

**Figure 9:**
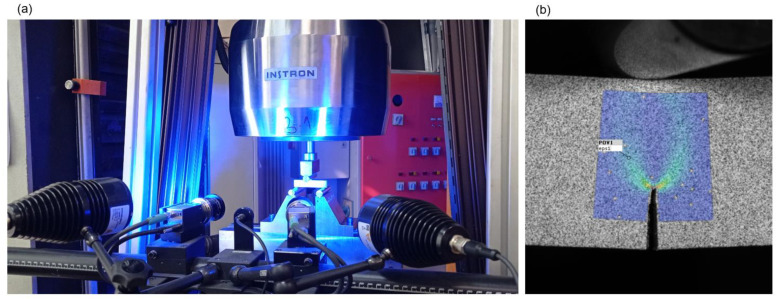
Fracture toughness testing of SENB specimen according to ASTM E1820: (**a**) test setup; (**b**) surface measurements with ARAMIS system.

After the fatigue precracking procedure is carried out, the fracture toughness measurements on three-point bending SENB specimens is performed according to ASTM E1820 standard. All tests are performed on a multipurpose servo-hydraulic testing machine INSTRON 1255 with crosshead displacement control at displacement rate 1.5 mm/min and at room temperature of 20 °C. The fixture system is equipped with two bottom fixed support rollers, with mutual span distance *S* = 58.7 mm, and top roller located at the specimen center, moving downwards under the vertical applied load *P* ≈ 9–17.5 kN, depending on the specimens’ resistance. The diameter of all rollers is *R* = 14 mm. Measurements on the surface of specimens are carried out using stereo-optical system ARAMIS, with fracture toughness test setup shown in Figure 9.

The purpose of ASTM E1820 fracture testing is to determine the fracture toughness using the characteristic parameters, applied load-CMOD curves, material fracture resistance in form of *J*-*R* curves and CTOD(*δ)*-*R* curves. Those parameters with corresponding measured values will be discussed in detail in the Section 4.

### 2.6. Fractography

After completed fracture mechanics testing of SENB specimens, the detailed fractographic analysis is carried out to provide deeper insight into crack behavior of specific welded joint zones. The macroscopic analysis of the cracked surfaces is performed using the Keyence VHX (Keyence Corp., Itasca, IL, USA) multifunctional stereoscopic microscope. 

## 3. Numerical Methods

### 3.1. Welded Joint Simplified Model

**Figure 10:**
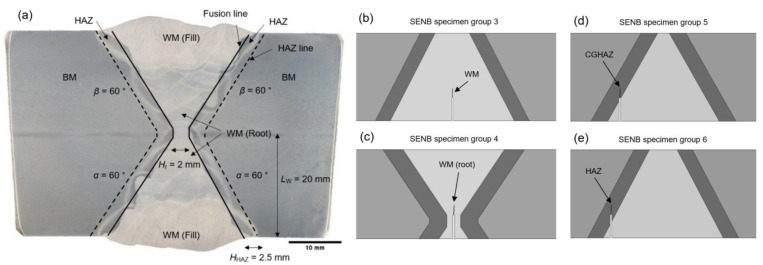
Simplification of X welded joint: (**a**) X welded joint macrograph with simplified fusion and HAZ lines; (**b**) SENB specimen group 3; (**c**) SENB specimen group 4; (**d**) SENB specimen group 5; (**e**) SENB specimen group 6.

Given the geometrical complexity and irregularity of welded joints in general, the need for simplified representation is mandatory for conducting numerical simulations. Existing fracture assessment procedures allow and idealization of actual weld geometry, where irregularities of the fusion lines, due to individual welding passes, can be represented only with straight lines. A methodology for simplification and idealized representation of V-groove weld has previously been proposed by Hertelé et al. [37]. The similar simplification principle in case of double-V weld (X weld) was used by Štefane et al. [15]. In continuation to mentioned simplification methodologies, for X welded joint examined in scope of this work, the authors also implemented bi-linear idealisation of fusion lines. In contrast to previous welded joint representations that included only BM and WM zones, this work also includes and examines the HAZ zone of welded joint. To model simplified HAZ, the fusion lines are being offset into the BM sides for the overall HAZ thickness value. Such general simplification of X welded joint is shown in Figure 10a. Simplifications of SENB specimens models, according to testing groups 3–6 and their corresponding disposition inside the welded joint, are shown in Figure 10b–e.

Simplification process is based on X joint macroscopic image with extracted dimensions of interest: root zone width *H*_r_ = 2 mm, HAZ width *H*_HAZ_ = 2.5 mm and weld center height *L*_W_ = 20 mm, with rounded values. The methodology used is similar to that previously mentioned by Štefane et al. [15]. The geometry simplification for all models is done with central vertical as well as a horizontal symmetry approach, using equal line angles *α* = *β* = 60°. This approach equalizes the top and bottom sides of the welded joint for further simplification. Shown in Figure 10a.

The exact SENB specimens positions and dimensions are done in accordance to extracted material volumes and sketches from Figure 7. Primary weld dimensions *H*_r_, *H*_HAZ_, *L*_W_ and *α* = *β* have the same values for all SENB specimens, since such simplification greatly facilitates modeling process, without significant adverse influence on simulation results. SENB specimen models for testing columnar WM on the weld centre line, upper and lower side of X joint (specimen group 3), WM root of lower side of X joint (specimen group 4), CGHAZ adjacent to columnar weld metal (specimen group 5) and through HAZ volume and the upper and lower side of the X joint (specimen group 6) are shown in Figure 10b–e.

### 3.2. Test Specimen Numerical Models

Numerical representation of SENB test specimen and test setup is prepared in two-dimensional environment. Such numerical representation is developed based on the SENB specimen, notch and crack modelling approaches, previously established by Starčevič et al. [8] and Štefane et al. [15]. As the reference indicates, the authors also used other approaches of numerical modelling SENB specimens and corresponding cracks, carried out by various researchers, described in the available literature [38,39,40,41,42]. All of the numerical simulations are carried out in Simulia Abaqus (2016, Dassault Systemes Simulia Corp., Johnston, RI, USA) finite element method software [43], using the explicit solver.

**Figure 11:**
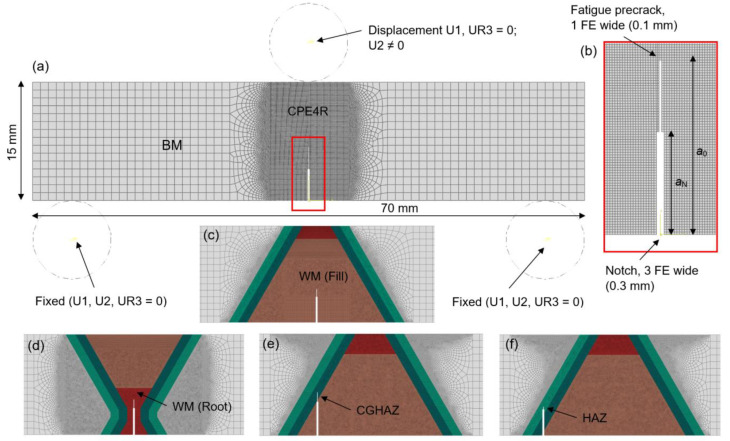
Finite element models of SENB specimens test setup: (**a**) three-point bending test setup; (**b**) notch and fatigue precrack; (**c**) SENB specimen group 3; (**d**) SENB specimen group 4; (**e**) SENB specimen group 5; (**f**) SENB specimen group 6.

The complete FE model of test setup consists of SENB specimen and three-point bending fixture system, as shown in Figure 11a. Two bottom fixed rollers (U1, U2, UR3 = 0), and one top roller are modelled as analytic rigid bodies, with diameter of *R* = 14 mm. Top roller has defined vertical displacement (U2 ≠ 0) over time, thus simulating the applied load *P*. Using 2D simplification, even with very fine mesh in the region of interest, the FE model computational time is significantly reduced compared to 3D model, giving accurate simulation results.

SENB specimen is modelled as a planar shell, completely discretised with a 4-node bilinear plane strain quadrilateral, first order, explicit FE CPE4R. The welded joint region has structured/free mesh with elements size of 0.1 mm. The element size is increased up to 1 mm in specimen lateral non influential regions, Figure 11a. Narrow notch has length of *a*_N_ and width of 3 FE, while fatigue precrack extends up to length of *a*_0_ and has width of 1 FE, as shown in Figure 11b.

In order to model the characteristic welded joint zones, the specimens are divided according to X welded joint simplification from Figure 10. The modelled zones are BM, WM (Fill), WM (Root), CGHAZ and HAZ with mechanical properties defined from MTS specimens testing and corresponding ductile damage parameters, shown in Figure 11c–f.

### 3.3. Ductile Damage

To describe the damage evolution capability of ductile metallic materials, the Ductile Damage material model [44] is implemented in numerical simulations carried out in Simulia Abaqus finite element method software [43]. The model assumes that damage is a function of progressive degradation of the material stiffness up to final material failure. It relies on Damage initiation criterion for ductile metals and is used complimentary with elasto–plastic material properties to describe the overall material behavior. Upon material failure, the removal of elements from the mesh takes effect.

**Figure 12:**
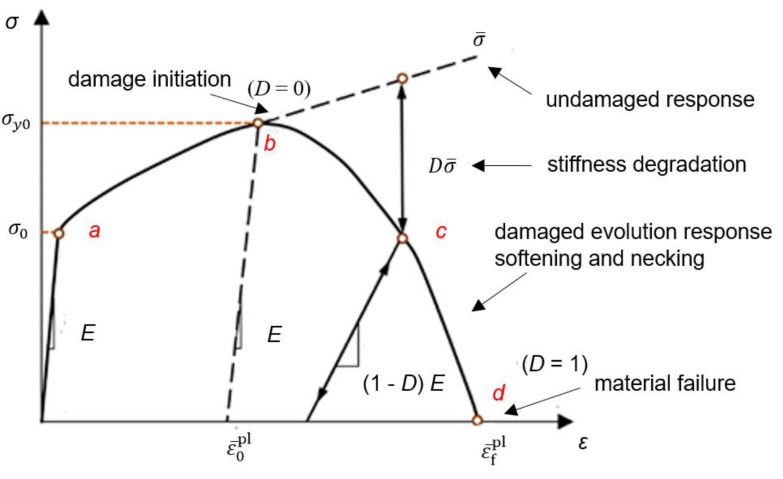
Stress–strain curve with progressive damage degradation [44].

The typical tensile test stress–strain curve, with progressive damage degradation is shown in Figure 12 [44]. Initial curve partition (0a) is linear-elastic part. Past the yield stress *σ*_0_, in curve partition (ab), the material undergoes stable plastic deformation with strain hardening effect. The point b is the initiation threshold of plastic instability, with damage parameter *D* = 0. In the failure partition (bd) stiffness degradation and damage evolution is present. At point d damage parameter *D* = 1, cracked material is present as an indication of failure [44].

The damage parameter *D*, governing the failure model, is defined by the equation:

(2) $$D=\sum \frac{{\bar{\varepsilon }}^{pl}}{{\bar{\varepsilon }}_{f}^{pl}}\leq 1$$

where ${\bar{\varepsilon }}^{pl}$ is equivalent plastic strain, and ${\bar{\varepsilon }}_{f}^{pl}$ is plastic strain at failure. Damage parameter D is changing from 0 (non-damaged) to 1 (material failure). At arbitrary time increment in the analysis, the damaged stress state is given by the scalar damage equation:

(3) $$\sigma_{D}=(1-D)\bar{\sigma }$$

where $\bar{\sigma }$ is the effective (undamaged) stress calculated in the current increment. For the ductile damage initiation, the model asumes that the equivalent plastic strain at the damage onset ${\bar{\varepsilon }}_{D}^{pl}$ is function of stress triaxiality *η* and equivalent plastic strain rate ${\dot{\bar{\varepsilon }}}^{pl}$:

(4) $${\bar{\varepsilon }}_{D}^{pl}\left(\eta ,{\dot{\bar{\varepsilon }}}^{pl}\right)$$

Damage evolution defines the post damage-initiation material behavior. The equivalent plastic displacement ${\bar{u}}^{pl}$, after damage initiation, is defined according to equation:

(5) $$\dot{{\bar{u}}^{pl}}=L\dot{{\bar{\varepsilon }}^{pl}}$$

where *L* is the characteristic mesh element length. Parameter ${\bar{u}}_{f}^{pl}$ relates to elongation of the element from damage initiation to failure. Severely damaged elements, reaching the maximum degradation limit, are deleted from the model thus achieving the specimen geometry separation. Asuming the constant stress triaxiality and strain rate, material damage behavior is strongly dependant on damage initiation fracture strain ${\bar{\varepsilon }}_{D}^{pl}$ parameter, and damage evolution displacement at failure ${\bar{u}}_{f}^{pl}$ parameter [44].

The ductile damage model proved to be generally suitable for modeling the behavior of HSS steels, which is evident from the research previously carried out by different authors. Ductile damage is used in numerical modelling of mismatched mechanical properties welded joint and corresponding SENB test specimens in research by Starčevič et al. [8]. Calibration of ductile damage model for HSS structural steels and tensile test simulations is carried out by Yang et al. [45]. Application of the ductile damage model in scope of numerical modeling of tensile and other steel test specimens is described in the literature [11,45,46,47,48,49].

**Figure 13:**
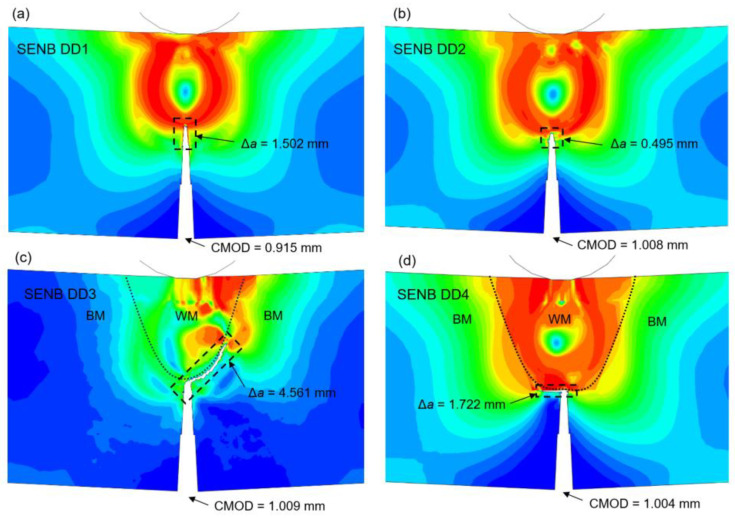
Damage behavior of SENB specimens during three-point bending tests: (**a**) SENB DD1; (**b**) SENB DD2; (**c**) SENB DD3; (**d**) SENB DD4.

The comparison of several SENB DD (Ductile Damage) specimens damage behavior, made from steel material, during three-point bending tests is shown in Figure 13. Ductile damage material and parameter variations are given in Table 8.

It can be observed that increase in fracture strain ${\bar{\varepsilon }}_{f}^{pl}$ parameter leads to increased material ductility, while displacement at failure ${\bar{u}}_{f}^{pl}$ affects the crack growth, as shown in homogenous BM specimens in Figure 13a,b. Crack growth following the WM/BM boundary, and crack arresting, dependant on welded joint geometry and material properties are shown in Figure 13c,d.

## 4. Results

The research covered in the paper is related to experimental testing and numerical analysis of fracture mechanics behavior inside the S690QL1 grade HSS heterogeneous welded joint zones. Experimental work is focused on tensile testing to obtain stress-strain properties throughout the welded joint, while fracture mechanics testing is used to determine the fracture toughness and all related resistance curves. Crack behavior is further investigated by means of fractographic analysis. Numerical simulations of SENB specimens fracture behavior are carried out using the ductile damage material model and further correlated to DIC measurements.

### 4.1. Mini Tensile Specimens (MTS) Testing

Characteristic values of tensile material behavior are determined through tensile testing of MTS specimens. Those values are yield strength (*R*_p0.2_), tensile strength (*R*_m_), percentage total elongation at maximum force (*A*_gt_), and Young’s modulus (*E*). For each specimen tested, the corresponding stress–strain (*σ*–*ε*) curve is constructed. Implementing the MTS specimens experimental methodology, the complete characterization of welded joint is possible. The stress–strain curves and tensile properties are determined for the fill passes, as well as root passes locations. 

Tensile testing along the welded joint transverse line in fill passes material volume is carried out using the MTS R1–R25 specimens, with results given in Table 9.

Tensile testing along the welded joint transverse line in root passes material volume is carried out using the MTS C1–C10 specimens, with results given in Table 10.

**Figure 14:**
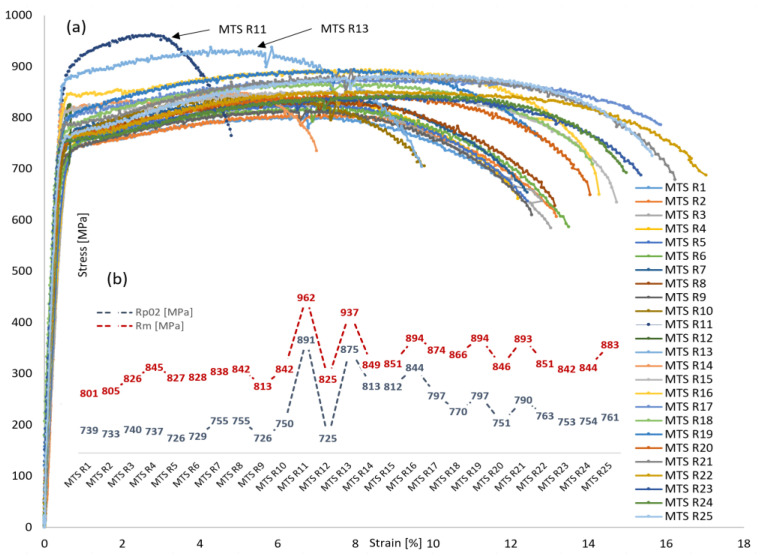
MTS testing of S690QL1 steel welded joint, fill passes volume: (**a**) stress–strain curves; (**b**) *R*_p0.2_/*R*_m_ strength values trendline.

Corresponding stress–strain curves obtained from MTS tensile tests for fill passes material volume are given in Figure 14a, with yield strength (*R*_p0.2_) and tensile strength (*R*_m_) values trendline shown in Figure 14b. The overall tensile strength values increase can be observed moving from BM to WM. In HAZ zone however, due to local microstructural changes, there is a trendline discontinuity, with two local peaks: MTS R11 (*R*_p0.2_ = 891 MPa and *R*_m_ = 962 MPa) and MTS R13 (*R*_p0.2_ = 875 MPa and *R*_m_ = 937 MPa). Stress-strain curves related to MTS R11 and R13 show certain deviation with ductility drop, having *A*_gt_ = 2.8% (MTS R11) and *A*_gt_ = 4.3% (MTS R13). The lowest tensile strength values are determined in BM at MTS R1 (*R*_p0.2_ = 739 MPa and *R*_m_ = 801 MPa), making this the weakest location in the fill passes transverse line.

**Figure 15:**
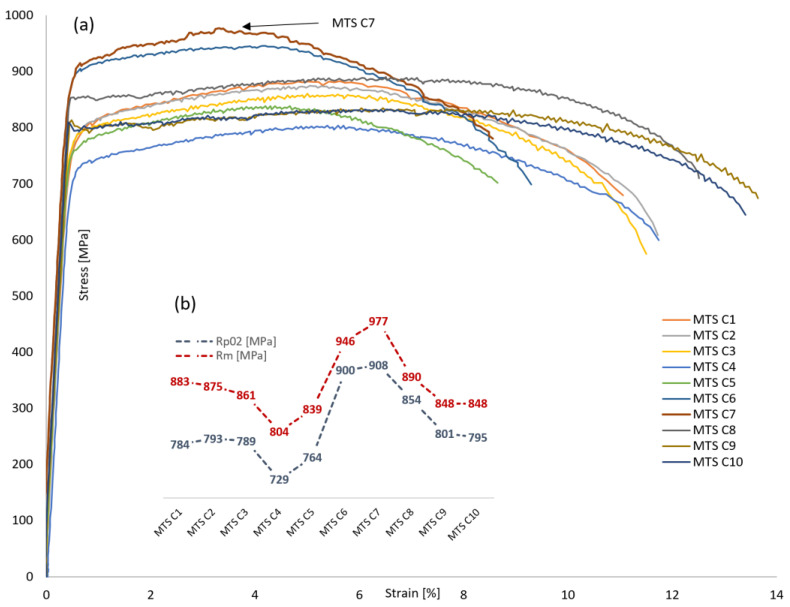
MTS testing of S690QL1 steel welded joint, root passes volume: (**a**) stress–strain curves; (**b**) *R*_p0.2_/*R*_m_ strength values trendline.

Corresponding stress-strain curves obtained from MTS tensile tests for root passes material volume are given in Figure 15a, with yield strength (*R*_p0.2_) and tensile strength (*R*_m_) values trendline shown in Figure 15b. The strength values are exhibiting mostly uniform trend in BM (MTS C1–C5) and WM (MTS C9 and C10). In BM zone, the minimum strength drop can be observed in MTS C4 (*R*_p0.2_ = 729 MPa and *R*_m_ = 804 MPa), making this the weakest location in root passes transverse line. HAZ zone is characterized by steep strength increase up to maximum peak in MTS C7 (*R*_p0.2_ = 908 MPa and *R*_m_ = 977 MPa).

**Figure 16:**
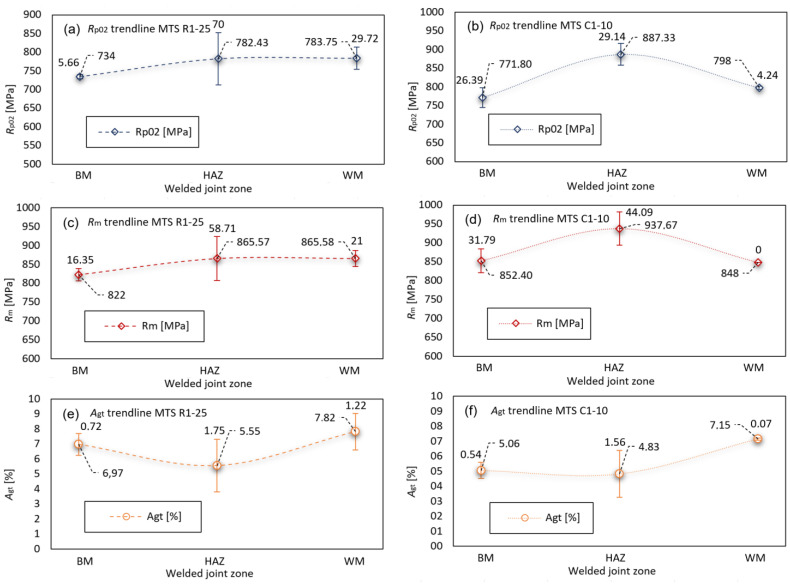
Statistical trendlines for MTS testing of S690QL1 steel welded joint: (**a**) fill passes *R*_p0.2_; (**b**) root passes *R*_p0.2_; (**c**) fill passes *R*_m_; (**d**) root passes *R*_m_; (**e**) fill passes *A*_gt_; (**f**) root passes *A*_gt_.

The stress–strain parameters *R*_p0.2_, *R*_m_ and *A*_gt_, according to welded joint zones, obtained through MTS testing, are further processed using statistical methods to determine the average values and standard deviation, as shown in Figure 16. It can be observed that average strength values *R*_p0.2_ and *R*_m_ are almost the same in HAZ and WM for fill passes (Figure 16a,c). In root passes, however, the *R*_p0.2_ and *R*_m_ have significant increase in HAZ (Figure 16b,d). Even though the HAZ is generally regarded as the weak zone in the welded joint, maintaining the low heat input during welding process of the S690QL1 X joint, the obtained HAZ fine grained zone mechanical properties are very high, and further strength increase is present in the coarse grained zone. This is in accordance with previous investigations of S690QL1 X welded joint, including hardness distribution transverse to welded joint, strength in specific weld zones and microstructural examinations, carried out by authors Tomerlin et al. [4]. Total elongation at maximum force *A*_gt_ exhibit the highest values in WM in both fill and root passes (Figure 16e,f). For all of the measurements, the highest deviation of results can be seen in the HAZ zone.

The experimentally determined results from tensile testing are further implemented into elasto-plastic material model with ductile damage, used in numerical simulations of SENB specimens fracture behavior, carried out in the scope of this paper.

### 4.2. Fracture Mechanics Testing—Fracture Toughness

**Figure 17:**
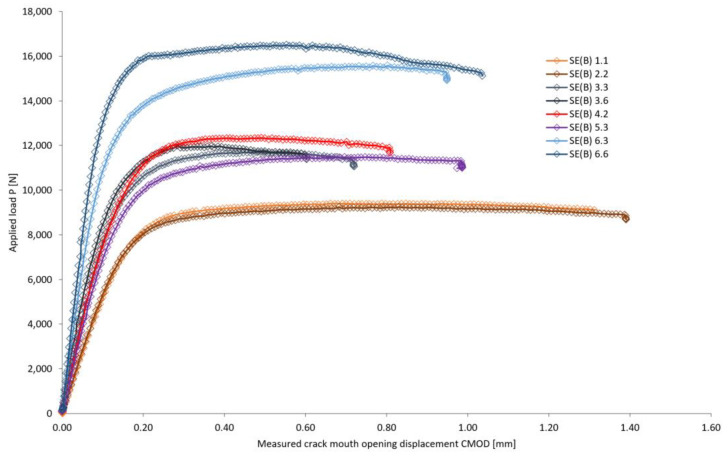
Applied load *P*-CMOD curves representative of each investigated welded joint zone.

In scope of fracture toughness testing of welded joint material, the measurements of applied load *P* vs. Crack Mouth Opening Displacement (CMOD) are recorded for all of the SENB specimens according to Figure 5 and Table 6. During the three-point bending of specimens, the CMOD displacement is measured on the surface of specimens. Maximum measured values of applied load *P*_max_ and CMOD_max_, for all of the tested specimens, are given in tabular form in Table 11. Several load-CMOD curves, representative of each welded joint zone and the corresponding SENB specimen groups, are shown in Figure 17.

Looking at the average applied load *P*_max_ and displacement CMOD_max_ values of specific welded joint zones, it is indicative that lowest resistance with large displacement is recorded in BM (*P*_avg_ = 9.35 kN, CMOD_avg_ = 1.08 mm), while highest resistance with large displacement is recorded in HAZ (*P*_avg_ = 14.55 kN, CMOD_avg_ = 1.13 mm). Other regions exhibit the following behavior: WM-Fill (*P*_avg_ = 11.73 kN, CMOD_avg_ = 0.70 mm), WM-Root (*P*_avg_ = 12.42 kN, CMOD_avg_ = 0.72 mm) and CGHAZ (*P*_avg_ = 10.27 kN, CMOD_avg_ = 1.18 mm). The welded joint material from the tested specimens, according to ASTM E1820 standard, exhibit blunting and stable crack growth.

According to ASTM E1820, the material fracture resistance in form of *J*-*R* curves and Crack Tip Opening Displacement CTOD(*δ)*-*R* curves is determined. The fracture testing characteristic values are determined, where *J*_Q(1)_ is *J*-Integral estimated value, *K*_JIC_ is Stress Intensity Factor (SIF) evaluated from *J*-Integral and *δ*_Q(1)_ is *δ* estimated value. Those values are recorded for all of the SENB specimens and given in tabular form in Table 12. The welded joint material, investigated in this work, undergoes ductile fracture with elastic–plastic fracture toughness measurements using *J*-integral or CTOD. However, the stress intensity factor *K* is often used to characterize the toughness of material that undergoes the brittle fracture. In scope of this work, the parameters *K*_JIC_ and *J*_IC_ are both given in order to provide the means of actual comparison of critical values.

The *J*-Δ*a* and *δ*-Δ*a* curves are constructed according to ASTM E1820 standard [35]. After plotting the *J* versus Δ*a* results, the construction line (i.e., blunting line) is determined from material tensile properties, as given in the equations:

(6) $$J=2\sigma_{Y}\Delta a$$

(7) $$\sigma_{Y}=\frac{\sigma_{YS}+\sigma_{TS}}{2}$$

where $\sigma_{Y}$ is the effective yield strength, $\sigma_{YS}$ is the yield strength (YS), and $\sigma_{TS}$ is the ultimate tensile strength (UTS). Parallel to the construction line, the offset lines are drawn, intersecting the abscissa at 0.15 mm, 0.20 mm, 0.50 mm and 2.00 mm. The regression line is determined using the linear least squares fitting function equation:

(8) $$lnJ=lnC_{1}+C_{2}ln\left(\frac{\Delta a}{k}\right)$$

where $C_{1}$ and $C_{2}$ are regression fit function coefficients and coefficient *k* = 1.0 mm. The power-law regression fit curve is determined from the following relationship:

(9) $$J_{Q(i+1)}=C_{1}{\left(\frac{{\Delta a}_{\left(i\right)}}{k}\right)}^{C_{2}}$$

Stress intensity factor (SIF) *K*_JIC_ can be evaluated from *J*_IC_ according to the following equation:

(10) $$K_{JIC}=\sqrt{\left(\frac{E}{\left(1-\nu^{2}\right)}\right)J_{IC}}$$

After plotting the *δ* versus Δ*a* results, the construction line (i.e., blunting line) is determined from material tensile properties, as given in the equation:

(11) $$\delta =1.4\sigma_{Y}\Delta a$$

Parallel to the construction line, the offset lines are drawn, intersecting the abscissa at 0.15 mm, 0.20 mm, 0.50 mm, and 2.00 mm. The regression line is determined using the linear least squares fitting function equation:

(12) $$ln\delta =lnC_{1}+C_{2}ln\left(\frac{\Delta a}{k}\right)$$

The power-law regression fit curve is determined from the following relationship:

(13) $$\delta_{Q(i+1)}=C_{1}{\left(\frac{{\Delta a}_{\left(i\right)}}{k}\right)}^{C_{2}}$$

Due to its complexity, only an outline of the *J*-integral and CTOD resistance curves development procedure is presented here. Detailed procedures are given in the ASTM E1820 standard.

*J*-integral estimated values *J*_Q_ are determined from *J*-*R* curves at the intersection point of regression line with 0.2 mm offset line, while estimated values *δ*_Q_ are determined from CTOD(*δ)*-*R* curves at the intersection point of regression line with 0.2 mm offset line. These values are given in Table 12. From the estimated values, using the validity requirements equations from the ASTM E1820, the qualification of *J*_Q_ as *J*_IC_ and *δ*_Q_ as *δ*_IC_ can be carried out. The qualification of all values in scope of SENB testing is valid except for those marked with *.

**Figure 18:**
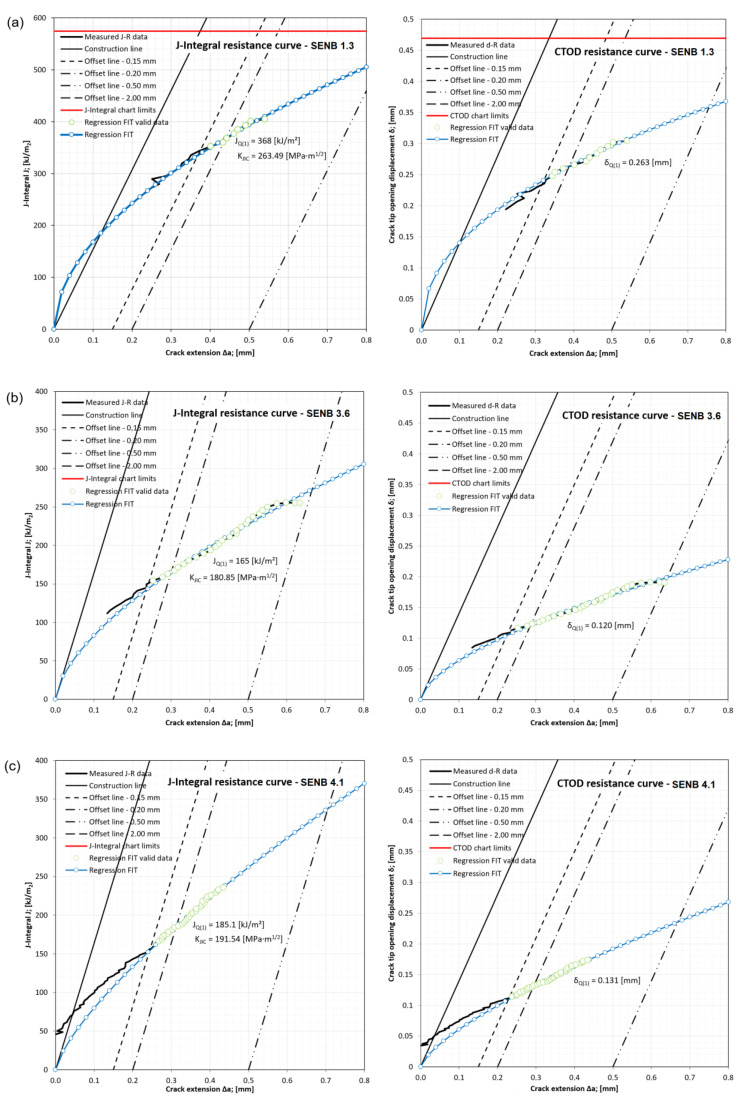

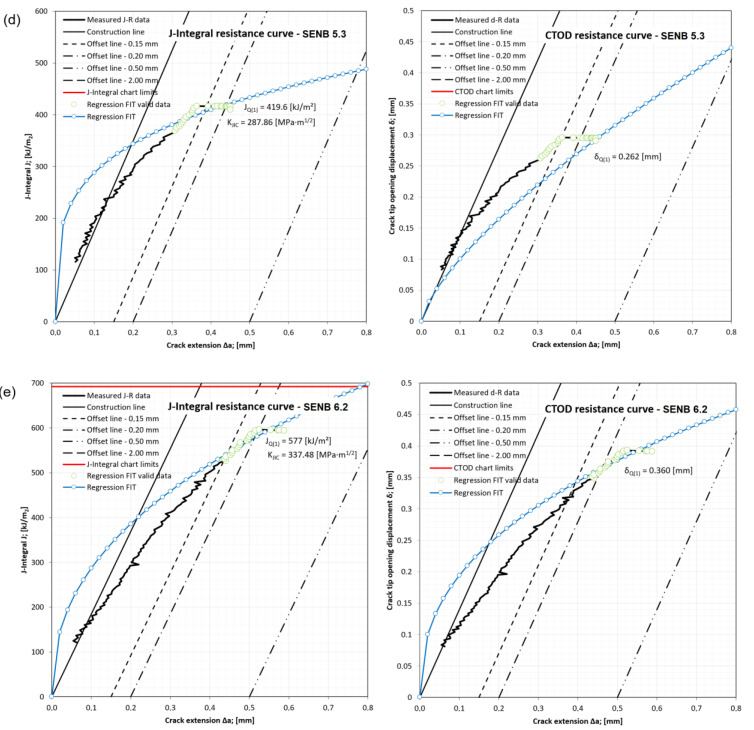
*J*-Integral resistance curves and CTOD resistance curves: (**a**) SENB 1.3 for groups 1 and 2; (**b**) SENB 3.6 for group 3; (**c**) SENB 4.1 for group 4; (**d**) SENB 5.3 for group 5; (**e**) SENB 6.2 for group 6.

After taking into account the average values of fracture toughness behavior in specific welded joint zones and corresponding SENB specimen groups, several *J*-*R* and CTOD(*δ)*-*R* representative curves are selected and shown in Figure 18. Specimen groups 1 and 2 for BM are represented by SENB 1.3, group 3 for WM (Fill) by SENB 3.6, group 4 for WM (Root) by SENB 4.1, group 5 for CGHAZ by SENB 5.3 and group 6 for HAZ by SENB 6.2.

**Figure 19:**
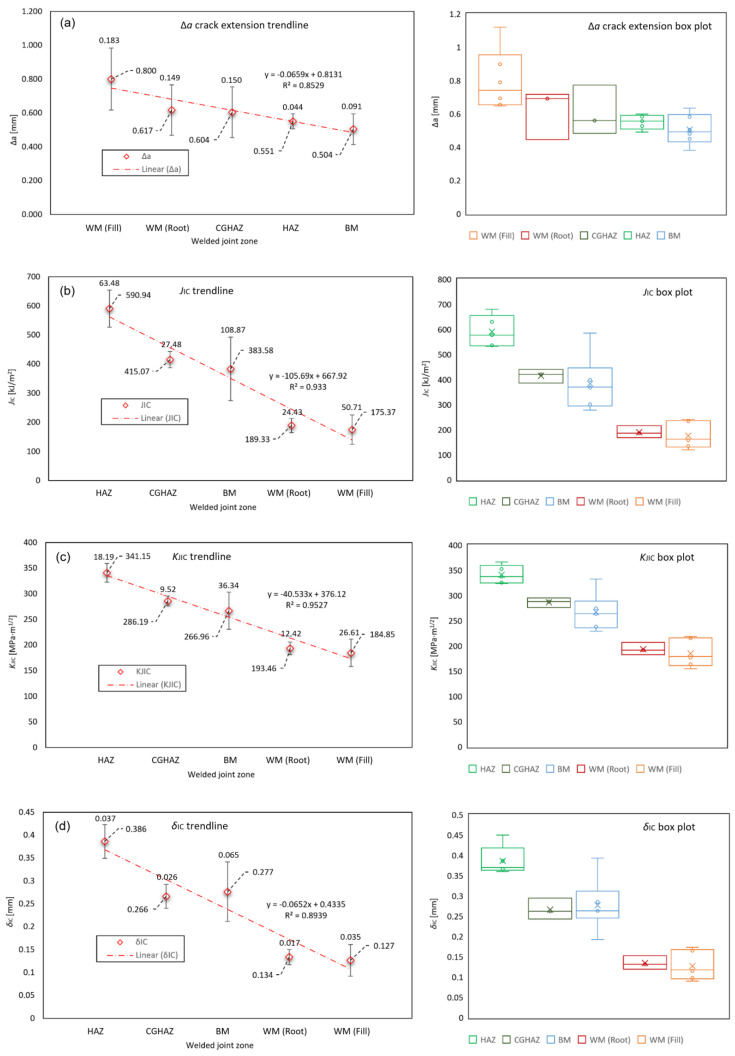
Statistical trendlines and box plots for fracture toughness parameters: (**a**) crack extension Δ*a*; (**b**) *J*-integral *J*_IC_; (**c**) stress intensity factor *K*_JIC_; (**d**) displacement *δ*_IC_.

Examining the fracture toughness testing parameters *J*_IC_, *K*_JIC_ and *δ*_IC_ trends throughout materials of different welded joint zones, based on averaged values, it can be concluded that, starting from the highest values, all of the values are: HAZ with *J*_IC_ = 590.940 kJ/m^2^, *K*_JIC_ = 341.146 MPa∙m^1/2^, *δ*_IC_ = 0.386 mm; CGHAZ with *J*_IC_ = 415.067 kJ/m^2^, *K*_JIC_ = 286.193 MPa∙m^1/2^, *δ*_IC_ = 0.266 mm; BM with *J*_IC_ = 383.583 kJ/m^2^, *K*_JIC_ = 266.962 MPa∙m^1/2^, *δ*_IC_ = 0.277 mm; WM (Root) with *J*_IC_ = 189.333 kJ/m^2^, *K*_JIC_ = 193.457 MPa∙m^1/2^, *δ*_IC_ = 0.134 mm; WM (Fill) with *J*_IC_ = 175.367 kJ/m^2^, *K*_JIC_ = 184.851 MPa∙m^1/2^, *δ*_IC_ = 0.127 mm. All values are given in Table 12. Crack extension Δ*a* trends, starting from the highest crack extension, values are: WM (Fill) with Δ*a* = 0.800 mm; WM (Root) with Δ*a* = 0.617 mm; CGHAZ with Δ*a* = 0.604 mm; HAZ with Δ*a* = 0.551 mm; BM with Δ*a* = 0.617 mm. All values are given in Table 7. Fracture toughness parameters Δ*a*, *J*_IC_, *K*_JIC_ and *δ*_IC_ comparative trendlines showing average values and standard deviation bars, as well as statistical box plots, according to welded joint zones, are shown in Figure 19. The slopes of individual linear regression lines indicate the rate of specific fracture parameters values drop, across the welded joint zones. The HAZ specimen 6.6 has significant values deviation, so it is excluded from statistical analysis.

Observing the fracture behavior of material, it is apparent that crack growth is most significant in WM (Fill) material, with largest crack extension and lowest fracture resistance values (Δ*a* = max.; *J*_IC_, *K*_JIC_, *δ*_IC_ = min.). The least crack growth can be observed in HAZ material (Δ*a* = 2nd min. after BM.; *J*_IC_, *K*_JIC_, *δ*_IC_ = max.). In general, the HAZ is considered the weak zone in the welded joint. However, the investigations carried out show much higher fracture toughness of HAZ zone compared to BM and WM, thus indicating higher resistance to crack growth (Figure 19). The welding process is carried out while maintaining the low heat input Q = 0.85–0.87 kJ/mm (Table 4) in order to achieve HAZ structure with predominantly very high mechanical properties, and narrow softened region in the intercritical and subcritical HAZ zones. The fatigue precrack tip of HAZ testing SENB specimens 6.1–6.6 managed to enter the fine-grained HAZ zone, having the best overall mechanical properties, and thus the highest fracture toughness and resistance to crack growth.

Previous experimental investigations of fracture toughness parameters for HSS S690QL steel welds, using the same filler material Mn3Ni1CrMo, are carried out by researchers Ismar et al. [50]. They have tested BM and WM zones only, obtaining lower values for BM *J*_IC_ = 201–238 kJ/m^2^ and *K*_JIC_ = 202–227 MPa∙m^1/2^; and for WM *J*_IC_ = 117–133 kJ/m^2^ and *K*_JIC_ = 164–175 MPa∙m^1/2^. Obtained *δ*_IC_ values are similar for BM *δ*_IC_ = 0.25–0.27 mm; and for WM *δ*_IC_ = 0.12–0.13 mm [46]. Fracture toughness of S690QL welded joints (BM with avg. *J*_IC_ = 428.571 kJ/m^2^, *K*_JIC_ = 305.571 MPa∙m^1/2^; WM-OM with avg. *J*_IC_ = 183 kJ/m^2^, *K*_JIC_ = 218 MPa∙m^1/2^; WM-UM with avg. *J*_IC_ = 327 kJ/m^2^, *K*_JIC_ = 289.5 MPa∙m^1/2^) is investigated by Štefane et al. [15]. Investigation of fracture behavior of other high strength steels and welds (e.g., S700MC and HY80) is described in literature [51,52].

### 4.3. Fractography

**Figure 20:**
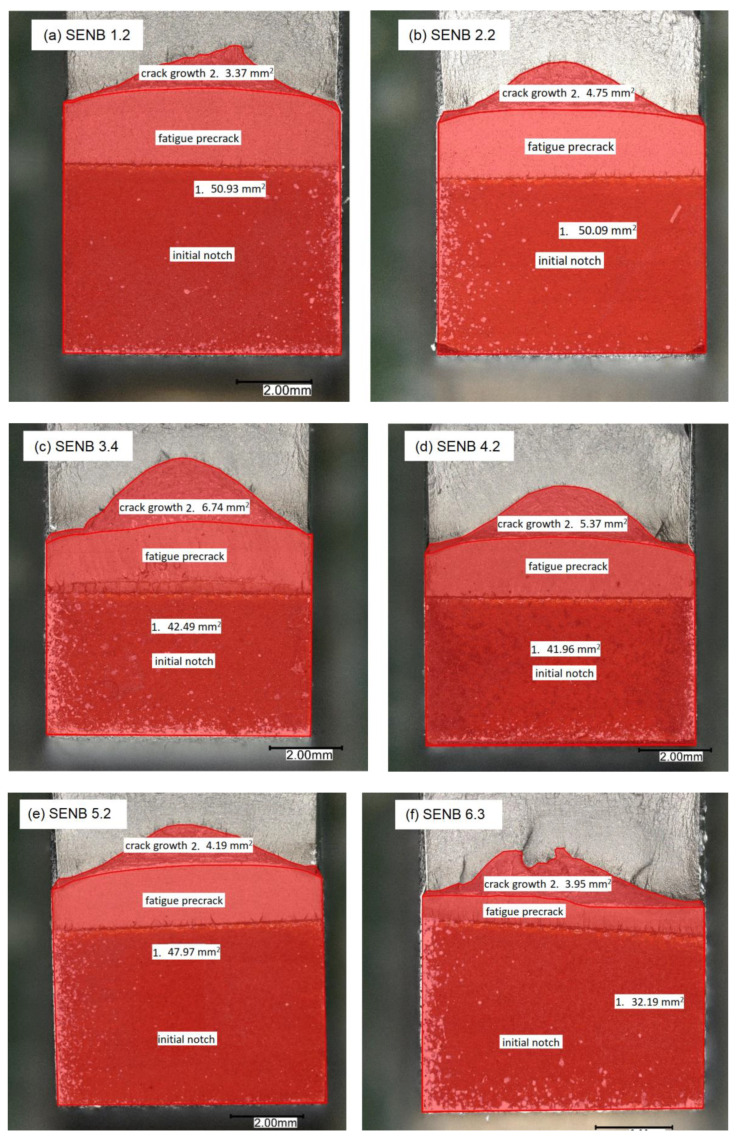
Fractographic images: (**a**) SENB 1.2 for group 1; (**b**) SENB 2.2 for group 2; (**c**) SENB 3.4 for group 3; (**d**) SENB 4.2 for group 4; (**e**) SENB 5.2 for group 5; (**f**) SENB 6.3 for group 6.

Fractographic examination of characteristic welded joint zones is carried out for several representative SENB specimens, with fracture surfaces images shown in Figure 20. On each fracture surface, the characteristic areas are visible and enumerated: 1. Initial notch + fatigue precrack area delimited with fatigue crack front and 2. Crack growth area delimited with the crack extension front. All specimens exhibit crack tunnelling, with only limited or no crack growth at all at specimen sides, in contrast to pronounced crack advancement in the center of specimen. The final ductile tearing area is visible on top section of each specimen. It can be observed that, according to welded joint zones, crack growth trend (area 2) starting from the smallest surface area is as follows: BM with SENB 1.2 (area 2 = 3.37 mm^2^), HAZ with SENB 6.3 (area 2 = 3.95 mm^2^), CGHAZ with SENB 5.2 (area 2 = 4.19 mm^2^), WM/Root with SENB 4.2 (area 2 = 5.37 mm^2^) and WM/Fill with SENB 3.4 (area 2 = 6.74 mm^2^).

### 4.4. Ductile Damage Material Parameters

As already mentioned, the characteristic welded joint zones of SENB specimens numerical models have associated corresponding elasto-plastic steel material properties with ductile damage evolution. In the scope of this research and this paper, the X welded joint is segmented into following zones of interest: BM, WM (Fill), WM (Root), CGHAZ, and HAZ, as previously shown in Figure 11c–f. In order to determine the elasto-plastic and damage material parameters for each of these zones, results from MTS tensile testing are used (as given in Section 4.1). Since in real material, due to various microstructural factors, the deviations of local mechanical properties are always inevitable, some material model calibrations are necessary to achieve a good numerical to experimental fit. As can be seen from the following fracture mechanics testing, the substantial variations of fracture mechanics properties between the SENB samples of the same representative groups, are also experimentally recorded. This is also an important factor during material models definition and numerical modelling of the SENB specimens’ behavior.

Elasto-plastic steel material parameters with included ductile damage for numerical analysis, according to X welded joint characteristic zones, and calibrated to specific SENB specimens, are given in Table 13.

Elasto-plastic steel material parameters *E*, *R*_p0.2_ and *R*_m_ for BM zone are calibrated from tensile tests MTS R1–R5 and MTS C1–C3, for WM (Fill) from tensile tests MTS R14–R25, for WM (Root) from tensile tests MTS C9–C10, for CGHAZ from tensile tests MTS R11–R13 and MTS C6–C8, and for HAZ from tensile tests MTS R6–R10 and MTS C4–C5. Poisson’s ratio ν = 0.33 is used for all material models. Ductile damage parameters are calculated and calibrated to describe the specific specimen’s behavior, per the methodology described in Section 3.3, and initial calibrations given in Table 8 and Figure 13. Ductile damage behavior includes the element deletion, with maximum element degradation specified at 0.95%.

### 4.5. Numerical Analysis

**Figure 21:**
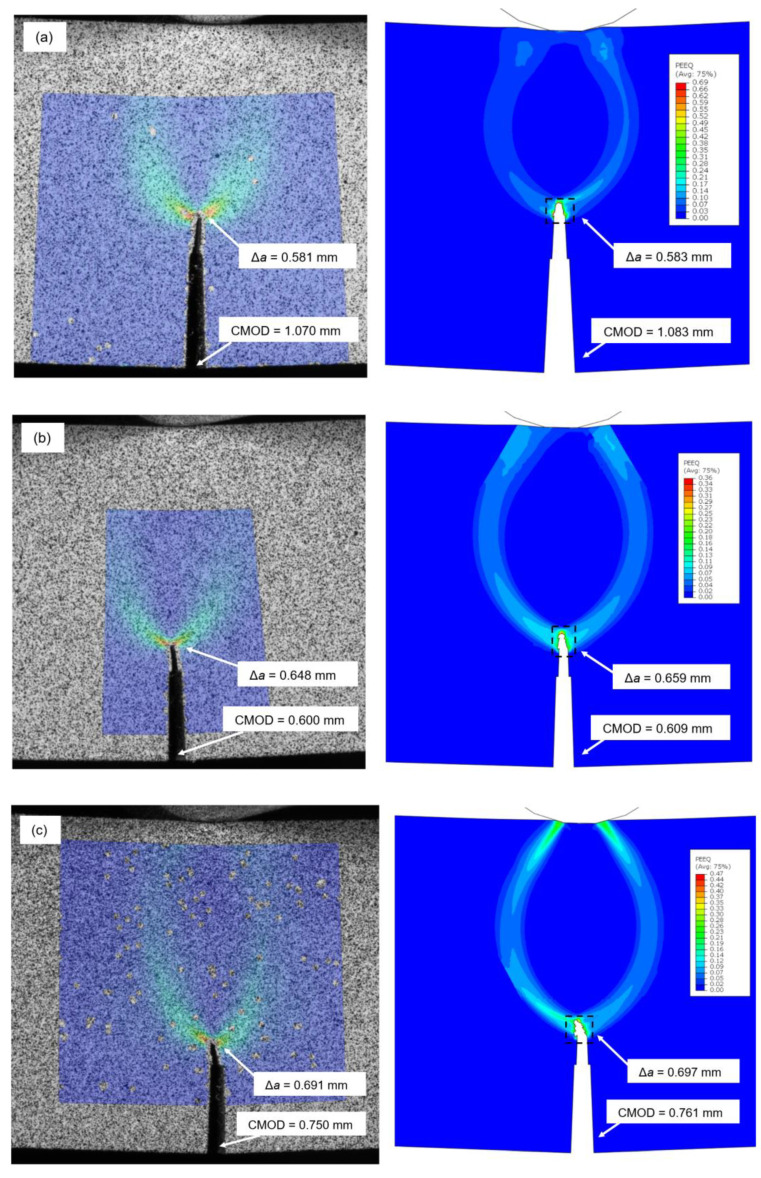

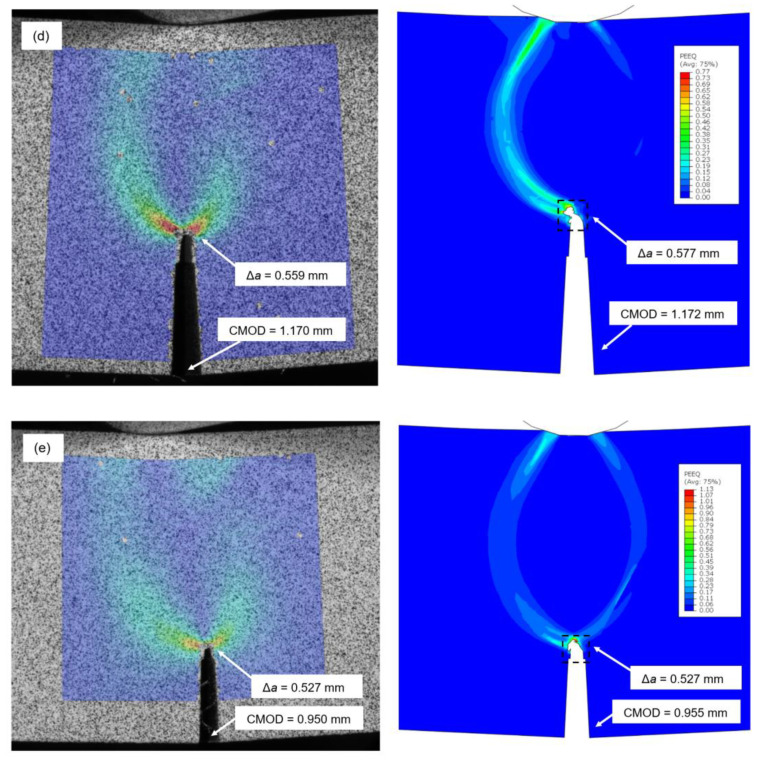
DIC vs. numerical analysis comparison—damage behavior of SENB specimens during three-point bending tests: (**a**) SENB 1.3 for groups 1 and 2; (**b**) SENB 3.6 for group 3; (**c**) SENB 4.3 for group 4; (**d**) SENB 5.2 for group 5; (**e**) SENB 6.3 for group 6.

The numerical analysis is carried out to describe the fracture behavior of SENB specimens, with simulated damage initiation and evolution, as accurately as possible compared to real ARAMIS measurements of samples employing DIC technique, shown in Figure 21.

The parameters used for comparison of crack behavior between DIC and numerically simulated SENB specimens, are crack extension Δ*a* and displacement CMOD, with determined values given in Table 14.

From the DIC vs. numerical analysis comparison images and determined fracture parameters values, the following observations can be made. For SENB specimen 1.3, representing BM material, crack extension is oriented in vertical upwards direction due to the homogeneity of the tested specimen material. Strain field is symmetrical, as shown in Figure 21a. For SENB specimen 3.6, representing WM (Fill) material, crack extension is also oriented in vertical upwards direction due to large volume of homogeneous WM (Fill) material, which is placed in the crack growth path. Strain field is symmetrical, as shown in Figure 21b. For SENB specimen 4.3, representing WM (Root) material, crack extension is oriented in vertical upwards direction with slight side deflection. WM (Root) material placed in the crack growth path is of smaller overall volume. The strain field is symmetrical, but with increased intensity in the crack deflection path, as shown in Figure 21c. For SENB specimen 5.2, representing CGHAZ material, crack extension is oriented in vertical, side deflected direction, corresponding to propagation through zones of lower material resistance. The strain field is unsymmetrical, with significantly increased intensity in the crack deflection path, as shown in Figure 21d. For SENB specimen 6.3, representing HAZ material, the crack extension is (similarly to previous CGHAZ case) oriented in a vertical, side deflected direction corresponding to propagation through zones of lower material resistance. The train field is slightly unsymmetrical, with moderately increased intensity in the crack deflection path as shown in Figure 21e. 

Analyzing and comparing the fracture parameters Δ*a* and CMOD, experimentally determined vs. numerically simulated, the percentage difference for all of the examined SENB specimens is very small (<3.17%), as shown in Table 14. Therefore, it can be concluded that numerical analysis shows good overall agreement with DIC measurements, which is evident based on all simulated SENB samples.

Since ductile damage material modelling and related methodology, used in previous numerical simulations, are not applicable for determining fracture toughness parameters *K*_IC_ and *J*_IC_, they will not be determined numerically in scope of this work. Suitable methods for this type of analysis, such as Contour integral or XFEM, are not used in this research.

## 5. Conclusions

In the scope of this research, the heterogeneity of welded joints and specific weld zones mechanical properties are investigated based on S690QL1 high strength steel material. Experimental work includes tensile testing and fracture mechanics testing. Tensile testing material properties are implemented into elasto-plastic material models with ductile damage used in numerical simulations of SENB specimens fracture behavior. Numerical simulations of SENB specimens fracture behavior are subsequently compared to experimentally measured results using the DIC technique. Upon completion of the research, the following conclusions could be drawn:

- Examining the crack extension Δ*a* trend throughout materials of different welded joint zones, based on averaged values, it can be concluded that, starting from the longest crack, crack extension lengths are: WM (Fill) with Δ*a* = 0.800 mm; WM (Root) with Δ*a* = 0.617 mm; CGHAZ with Δ*a* = 0.604 mm; HAZ with Δ*a* = 0.551 mm; BM with Δ*a* = 0.504 mm. All values are given in Table 7.
- Examining the fracture toughness testing parameters *J*_IC_, *K*_JIC_ and *δ*_IC_ trends throughout materials of different welded joint zones, based on averaged values, it can be concluded that, starting from the highest values, all of the values are: HAZ with *J*_IC_ = 590.940 kJ/m^2^, *K*_JIC_ = 341.146 MPa∙m^1/2^, *δ*_IC_ = 0.386 mm; CGHAZ with *J*_IC_ = 415.067 kJ/m^2^, *K*_JIC_ = 286.193 MPa∙m^1/2^, *δ*_IC_ = 0.266 mm; BM with *J*_IC_ = 383.583 kJ/m^2^, *K*_JIC_ = 266.962 MPa∙m^1/2^, *δ*_IC_ = 0.277 mm; WM (Root) with *J*_IC_ = 189.333 kJ/m^2^, *K*_JIC_ = 193.457 MPa∙m^1/2^, *δ*_IC_ = 0.134 mm; WM (Fill) with *J*_IC_ = 175.367 kJ/m^2^, *K*_JIC_ = 184.851 MPa∙m^1/2^, *δ*_IC_ = 0.127 mm. All values are given in Table 12.
- Fractographic examination shows the crack growth trend (area 2/Figure 19) starting from the smallest surface area: BM (area 2 = 3.37 mm^2^), HAZ (area 2 = 3.95 mm^2^), CGHAZ (area 2 = 4.19 mm^2^), WM/Root (area 2 = 5.37 mm^2^) and WM/Fill (area 2 = 6.74 mm^2^). This is shown in Figure 20.
- Overall fracture behavior of welded joint materials is determined, with crack growth being most significant in WM (Fill) material with largest crack extension and lowest fracture resistance values (Δ*a* = max.; *J*_IC_, *K*_JIC_, *δ*_IC_ = min.) and least significant in HAZ material (Δ*a* = 2nd min. after BM.; *J*_IC_, *K*_JIC_, *δ*_IC_ = max.).
- DIC vs. numerical analysis comparison of the SENB specimens fracture behavior shows good agreement, both visible qualitative on the crack extension orientation and quantitative comparing the crack extension Δ*a* and displacement CMOD parameters values. As seen in Figure 21 and Table 14.
- Elasto-plastic material model with ductile damage is able to describe the fracture behavior of heterogeneous material volumes, e.g., welded joints. Crack dimensions, growth and direction can be accurately modelled through input of ductile damage material parameters: fracture strain ${\bar{\varepsilon }}_{D}^{pl}$ and damage evolution displacement at failure ${\bar{u}}_{f}^{pl}$. The comparison of several SENB DD (Ductile Damage) specimens damage behavior, with different material parameters, is shown in Figure 13.

## Figures and Tables

**Table 1:** Welded joint HAZ zones properties [1].

HAZ Zone	Temperature Range	Mechanical Properties
CGHAZ	*T*_γδ_ < *T* < *T*_m_	Low toughness, high strength
FGHAZ	A_c3_ < *T* < *T*_γδ_	Adequate toughness and strength
ICHAZ	A_c1_ < *T* < A_c3_	High toughness, low strength
SCHAZ	A_c1_ < *T*	High toughness, low strength

*T* = welding process temperature; *T*_m_ = steel melting point; *T*_γδ_ = *γ* to *δ* transformation completed temperature; A_c3_ = *α* to *γ* transformation completed, upper critical temperature; A_c1_ = *α* to *γ* transformation started, lower critical temperature.

**Table 2:** Mechanical properties of S690QL1 and Mn3Ni1CrMo [24,25,26].

Material	Location	*R*_p0.2_	*R*_m_	*A*_5_	ISO-V ^2^	*M*
		[MPa]	[MPa]	[%]	[J]	
S690QL1 ^1^	BM	787	841	22	108 (−60 °C)	1
Mn3Ni1CrMo	WM	800	900	19	190 (20 °C)	1.16 (OM)

*R*_p0.2_: yield strength; *R*_m_: tensile strength; *A*_5_: percentage elongation after fracture; ISO-V: Charpy V-notch impact strength. ^1^ valid for 40 mm plate thickness, ^2^ transverse oriented specimen.

**Table 3:** Chemical composition of S690QL1 and Mn3Ni1CrMo [24,25,26].

Material	C	Si	Mn	Ni	Cr	Mo	Cu	Al	V	P	S	CEV ^1^	CET ^2^
	Chemical Element [% by Weight]												
S690QL1	0.17	0.25	1.20	0.05	0.33	0.21	0.03	0.075	0.01	0.011	0.001	0.49	0.33
Mn3Ni1CrMo	0.10	0.53	1.60	1.40	0.32	0.269	0.02	0.005	0.075	0.012	0.006	0.59	0.34

^1^ CEV = C + Mn/6 + (Cr + Mo + V)/5 + (Cu + Ni)/15—carbon equivalent according to International Institute of Welding (i.e., *CE*_IIW_); ^2^ CET = C + (Mn + Mo)/10 + (Cr + Cu)/20 + Ni/40—carbon equivalent according to EN 1011-2 [29].

**Table 4:** Welding process parameters.

Pass No.	Location	*I*	*U*	v_w_	*η*	Q	Δ*t*_8/5_
		[A]	[V]	[cm/min]		[kJ/mm]	[s]
1–3	Root	195	26	28.5	0.8	0.85	4.4
4–22	Fill + Cover	280	29	45	0.8	0.87	4.5

**Table 5:** MTS specimens positions inside welded joint.

MTS Specimen	Location	Welded Joint Zone	MTS Specimen	Location	Welded Joint Zone
R1–R6	Fill + Cover	BM	C1–C5	Root	BM
R7–R13	Fill + Cover	HAZ	C6–C8	Root	HAZ
R14–R25	Fill + Cover	WM	C9–C10	Root	WM

**Table 6:** SENB specimens positions inside welded joint.

SENB Specimen	Welded Joint Zone	Orientation	Crack
1.1–1.3	BM	NP	Crack initiation and through BM
2.1–2.3	BM	NP	Crack initiation and through BM
3.1–3.6	WM (Fill)	NQ	Crack initiation and through columnar WM, weld centre line, upper + lower side
4.1–4.3	WM (Root)	NQ	Crack in WM root, lower side propagation
5.1–5.3	CGHAZ	NQ	Crack initiation in CGHAZ, adjacent to WM
6.1–6.6	HAZ	NQ	Crack initiation in HAZ, upper + lower side

**Table 7:** SENB specimens fatigue precracking specifications and crack dimensions.

SENB Specimen	Location	*a*_0_	*a*_0_/*W*	*a*_p_	*a*_p_/*W*	Δ*a*
		[mm]	[-]	[mm]	[-]	[mm]
1.1	BM	7.162	0.478	7.666	0.512	0.504
1.2	BM	6.791	0.454	7.240	0.484	0.449
1.3	BM	6.824	0.456	7.405	0.495	0.581
2.1	BM	7.020	0.468	7.499	0.500	0.479
2.2	BM	6.679	0.446	7.312	0.488	0.633
2.3	BM	6.815	0.455	7.195	0.481	0.380
3.1	WM (Fill)	6.001	0.400	6.693	0.446	0.692
3.2	WM (Fill)	5.421	0.362	6.541	0.437	1.120
3.3	WM (Fill)	5.685	0.380	6.340	0.424	0.655
3.4	WM (Fill)	5.665	0.378	6.564	0.438	0.899
3.5	WM (Fill)	4.976	0.332	5.765	0.385	0.789
3.6	WM (Fill)	5.213	0.348	5.861	0.392	0.648
4.1	WM (Root)	5.941	0.396	6.387	0.426	0.445
4.2	WM (Root)	5.595	0.374	6.311	0.422	0.716
4.3	WM (Root)	5.295	0.354	5.985	0.400	0.691
5.1	CGHAZ	7.072	0.471	7.844	0.523	0.772
5.2	CGHAZ	6.396	0.427	6.955	0.465	0.559
5.3	CGHAZ	5.703	0.381	6.185	0.413	0.483
6.1	HAZ	6.060	0.404	6.549	0.437	0.489
6.2	HAZ	5.931	0.396	6.529	0.436	0.599
6.3	HAZ	4.292	0.293	4.819	0.328	0.527
6.4	HAZ	4.907	0.328	5.489	0.367	0.583
6.5	HAZ	3.793	0.253	4.349	0.291	0.556
6.6	HAZ	3.753	0.251	5.260	0.351	1.507

**Table 8:** Ductile damage parameters variations, SENB DD specimens.

Specimen	Material	*E*	*R*_p0.2_	*R*_m_	${\bar{\varepsilon }}_{f}^{pl}$	${\bar{u}}_{f}^{pl}$	Δ*a*	CMOD
		[GPa]	[MPa]	[MPa]		[mm]	[mm]	[mm]
SENB DD1	BM	210	755	840	0.075	0.030	1.502	0.915
SENB DD2	BM	210	755	840	0.075	0.055	0.495	1.008
SENB DD3	BM/WM	210/210	755/780	840/890	0.075/0.110	0.030/0.055	4.561	1.009
SENB DD4	BM/WM	210/210	690/780	770/890	0.090/0.110	0.025/0.200	1.722	1.004

**Table 9:** Tensile testing properties of S690QL1 steel welded joint, fill passes material volume.

Specimen	Location	*R*_p0.2_	*R*_m_	*E*	*A*_gt_
		[MPa]	[MPa]	[GPa]	[%]
MTS R1	BM	739	801	169.94	7.3
MTS R2	BM	733	805	148.84	8.1
MTS R3	BM	740	826	137.98	6.1
MTS R4	BM	737	845	131.31	6.3
MTS R5	BM	726	827	148.39	6.9
MTS R6	BM	729	828	164.30	7.1
MTS R7	HAZ	755	838	169.10	7.2
MTS R8	HAZ	755	842	188.26	7.3
MTS R9	HAZ	726	813	193.22	6.2
MTS R10	HAZ	750	842	161.03	5.5
MTS R11	HAZ	891	962	191.49	2.8
MTS R12	HAZ	725	825	-	-
MTS R13	HAZ	875	937	221.51	4.3
MTS R14	WM	813	849	186.16	4.9
MTS R15	WM	812	851	180.00	6.4
MTS R16	WM	844	894	218.40	8.2
MTS R17	WM	797	874	216.09	8.2
MTS R18	WM	770	866	176.20	6.8
MTS R19	WM	797	894	174.51	8.0
MTS R20	WM	751	846	176.07	7.9
MTS R21	WM	790	893	200.73	7.9
MTS R22	WM	763	851	186.36	8.8
MTS R23	WM	753	842	185.10	9.0
MTS R24	WM	754	844	213.36	8.9
MTS R25	WM	761	883	199.68	8.8

**Table 10:** Tensile testing properties of S690QL1 steel welded joint, root passes material volume.

Specimen	Location	*R*_p0.2_	*R*_m_	*E*	*A*_gt_
		[MPa]	[MPa]	[GPa]	[%]
MTS C1	BM	784	883	165.71	5.6
MTS C2	BM	793	875	180.17	5.1
MTS C3	BM	789	861	181.72	5.0
MTS C4	BM	729	804	171.69	5.4
MTS C5	BM	764	839	168.02	4.2
MTS C6	HAZ	900	946	179.62	4.6
MTS C7	HAZ	908	977	174.44	3.4
MTS C8	HAZ	854	890	179.71	6.5
MTS C9	WM	801	848	168.20	7.2
MTS C10	WM	795	848	180.37	7.1

**Table 11:** SENB specimens fracture toughness testing *P*_max_ and CMOD_max_ values.

SENB Specimen	Welded Joint Zone	*P*_max_	CMOD_max_
		[kN]	[mm]
1.1–1.3	BM	9.40; 8.89; 9.34	1.31; 1.02; 1.07
2.1–2.3	BM	9.85; 9.25; 9.38	0.75; 1.39; 0.92
3.1–3.6	WM (Fill)	11.98; 11.90; 11.70; 10.48; 12.34; 12.00	0.78; 0.82; 0.72; 0.74; 0.56; 0.60
4.1–4.3	WM (Root)	12.02; 12.34; 12.91	0.60; 0.81; 0.75
5.1.-5.3	CGHAZ	9.43; 9.89; 11.49	1.39; 1.17; 0.99
6.1–6.6	HAZ	11.92; 11.14; 15.59; 14.65; 17.50; 16.51	1.24; 1.38; 0.95; 1.25; 0.89; 1.04

**Table 12:** SENB specimens fracture toughness testing *J*_Q(1)_, *K*_JIC_ and *δ*_Q(1)_ values.

SENB Specimen	Welded Joint Zone	*J*_Q(1)_/*J*_IC_	*K*_JIC_	*δ*_Q(1)_/*δ*_IC_
		[kJ/m^2^]	[MPa∙m^1/2^]	[mm]
1.1–1.3	BM	585.00 *; 371.00; 368.00	332.22; 264.57; 263.49	0.393; 0.263; 0.263
2.1–2.3	BM	278.00; 399.00; 300.50	229.02; 274.37; 238.11	0.193; 0.285; 0.263
3.1–3.6	WM (Fill)	233.70; 158.00; 241.00; 134.50; 120.00; 165.00	215.23; 176.97; 218.56; 163.28; 154.23; 180.85	0.165; 0.115; 0.173; 0.097; 0.090; 0.120
4.1–4.3	WM (Root)	185.10; 215.60; 167.30	191.54; 206.72; 182.10	0.131; 0.152; 0.119
5.1–5.3	CGHAZ	385.60; 440.00; 419.60	275.95; 294.77; 287.86	0.243; 0.294; 0.262
6.1–6.6	HAZ	629.70; 577.00; 536.00; 680.00; 532.00; 182.00	352.56; 337.48; 325.27; 366.37; 324.05; 189.54	0.386; 0.360; 0.366; 0.450; 0.370; 0.127

* Qualification of *J*_Q_ as *J*_IC_ not valid.

**Table 13:** Material parameters according to welded joint zones, SENB specimens.

Specimen	Material	*E*	*R*_p0.2_	*R*_m_	${\bar{\varepsilon }}_{f}^{pl}$	${\bar{u}}_{f}^{pl}$
		[GPa]	[MPa]	[MPa]		[mm]
1.3	BM	210	735	820	0.119	0.054
3.6	BM	210	735	820	0.119	0.054
	WM (Fill)	210	780	865	0.146	0.024
	WM (Root)	210	800	850	0.134	0.032
	CGHAZ	210	860	925	0.078	0.038
	HAZ	210	745	830	0.123	0.044
4.3	BM	210	735	820	0.119	0.054
	WM (Fill)	210	780	865	0.149	0.028
	WM (Root)	210	800	850	0.139	0.033
	CGHAZ	210	860	925	0.078	0.038
	HAZ	210	745	830	0.123	0.044
5.2	BM	210	735	820	0.162	0.068
	WM (Fill)	210	780	865	0.149	0.028
	WM (Root)	210	800	850	0.139	0.033
	CGHAZ	210	860	925	0.128	0.029
	HAZ	210	745	855	0.164	0.073
6.3	BM	210	735	820	0.162	0.068
	WM (Fill)	210	780	865	0.149	0.028
	WM (Root)	210	800	850	0.139	0.033
	CGHAZ	210	860	925	0.128	0.029
	HAZ	210	745	855	0.177	0.087

**Table 14:** Fracture parameters of SENB specimens, DIC vs. numerical comparison.

Specimen	Material	Δ*a*_(EXP)_	Δ*a*_(NUM)_	Δ*a*_(diff.)_	CMOD_(EXP)_	CMOD_(NUM)_	CMOD_(diff.)_
		[mm]	[mm]	[%]	[mm]	[mm]	[%]
1.3	BM	0.581	0.583	0.34	1.070	1.083	1.21
3.6	WM (Fill)	0.648	0.659	1.68	0.600	0.609	1.49
4.3	WM (Root)	0.691	0.697	0.86	0.750	0.761	1.46
5.2	CGHAZ	0.559	0.577	3.17	1.170	1.172	0.17
6.3	HAZ	0.527	0.527	0	0.950	0.955	0.52

## Data Availability

Not applicable.
